# Phase separation in epigenetics and cancer stem cells

**DOI:** 10.3389/fonc.2022.922604

**Published:** 2022-08-23

**Authors:** Chanchan Xiao, Guangjie Wu, Pengfei Chen, Lijuan Gao, Guobing Chen, Hongyi Zhang

**Affiliations:** ^1^ Department of Microbiology and Immunology, School of Medicine, Jinan University, Guangzhou, China; ^2^ Institute of Geriatric Immunology, School of Medicine, Jinan University, Guangzhou, China; ^3^ Guangdong-Hongkong-Macao Great Bay Area Geroscience Joint Laboratory (GBGJL), School of Medicine, Jinan University, Guangzhou, China

**Keywords:** epigenetic, stemness, cancer stem cells, liquid-liquid phase separation, phase separation

## Abstract

Accumulating evidence indicates that liquid–liquid phase separation (LLPS) is the basis of the formation of membrane-less compartments in cells. This biomolecular condensate represented by phase separation may influence epigenetics in cancer stem cells (CSCs), a small subpopulation of cancer cells responding to the initiation, maintenance, metastasis, and therapy resistance of cancer. Understanding the underlying biophysical principles and the specific characteristics of biocondensates would provide insights into the precise blocking of potential tumor targets, thereby fundamentally curbing tumor occurrence, recurrence and metastasis. In this review, we summarized the key phenomenon and experimental detection of phase separation and the possibility of regulating the stemness of CSCs through phase separation. We believe that the mechanism of phase separation in CSCs will open up new avenues for the mystery of tumor formation, and modulating phase separation will be a great strategy for CSC-targeted tumor therapy.

## Introduction

The liquid compartments for liquid–liquid phase separation (LLPS) are spherical in shape, with fusion and fission phenotypes, and exhibit high internal mobility and high rates of exchange with the surrounding environment (1). For a long time, scientists have observed that biomacromolecules in cells are not distributed uniformly in the aqueous environment but instead form distinct compartments in the nucleus, cytoplasm or cell membrane. Cells use these compartments to organize cellular biochemical reactions, making these processes relatively independent, highly ordered, and efficient. Membrane-bound organelles use phospholipid bilayers to encapsulate intracellular biomolecules. There are also numerous nonmembrane-bound compartments in eukaryotic cells, postsynaptic densities in synapses, and protein spots at DNA damage repair sites (2, 3). According to recent studies, these nonmembrane-bound compartments share a similar assembly mechanism, termed liquid–liquid phase separation, and are collectively named biomolecular condensates. The phase-separated compartments further undergo a phase transition to specify their distinct material properties (4). The interactions of these phase-separated multivalent domains are abundant not only in the cytoplasm but also in epigenetic processes, such as the recognition of epigenetic modifications by their readers and the interaction between the complex composed of transcription factors and DNA sequences. Brangwynne’s group applied the CasDrop system to study three studied proteins (FUS, BRD4, and TAF15) forming the ‘LLPS’ phenomenon, which accompanied the sense and restructures of the genome in the nucleus (5).

Phase separation is widely associated with the development and disease (6). Although LLP research is still in its infancy, it is advancing rapidly, and it is clear that LLPS plays a crucial role in the development of pathophysiological conditions. The underlying mechanisms for LLPS are the regulation of transcription (7), genome organization (8), immune responses (9), and neuronal synaptic signaling (10), specifically in cancer (11), neurodegenerative diseases (12), and COVID-19 (13, 14). In 2009, Brangwynne’s group discovered that the RNA in *Caeno-rhabditis elegans* embryos and the protein-containing conjugate P granules were agglomerated spheres formed by protein phase separation (15). In 2011, another study also found that a dense cluster of genetic materials and proteins had droplet-like behaviors in the cell nucleus. Phase separation has important applications in organisms, such as the aggregation of proteins and nucleic acids involved in gene regulation, RNA processing, and other life processes (16). In biological applications, it is beneficial for the fabrication of nanofibrous scaffolds, which have interconnected porous structures. The latter facilitates cell migration, nutrient/waste exchange, and uniform cell and nutrient distribution (17). In addition, the phase separation beneficially allows the fabrication of interconnected porous scaffolds with complex geometries (17). Thus, phase separation is a valuable method for nanofibrous scaffold preparation for bone tissue engineering applications. It also shows significant implications in designing small molecule compounds to modulate the entry of functionally important guest molecules into aggregates for phase separation. Fang et al. identified some compounds that could block the entry of TDP-43 into stress granules intracellularly and thereby inhibit its accumulation of TDP-43 protein in neurons in amyotrophic lateral sclerosis with frontotemporal dementia (ALS/FTD) lesions (18).

It is interesting to investigate the roles of phase separation in regulating the characteristics of cancer stem cells (CSCs) and explore a new strategy for the treatment of tumors. CSCs were first identified in leukemia in 1997 (19) and subsequently found in breast cancer in 2003 (20). Leukemic stem cells have been shown to display the CD34+CD38− surface marker phenotype, in which the loss of CD38 distinguishes these cells from normal hematopoietic stem cells (19). Al-Hajj et al. (7) demonstrated that CD44+CD24−/(low) Lineage− cells isolated in eight of nine patients with breast cancer had the capacity to form tumors when serially transplanted into immunocompromised mice (20). CSCs have been identified in other solid tumors, including brain tumors, lung cancer, colon cancer, and melanoma (21–23). CSCs can self-renew, give rise to progeny that are different from them, and utilize common signaling pathways (24). Cancer stem cells may be the source of all the tumor cells present in a malignant tumor, the reason for the resistance to the chemotherapeutic agent used to treat the malignant tumor, and the source of cells that give rise to distant metastases (25). Briefly, CSCs are believed to be an important target for novel anticancer therapeutics (26). The source of CSCs is thought to be mutation of stem cells (CSCs originate from differentiated malignant cells that reactivated stem-like features leading to de-differentiation) or reprogramming of somatic cells by genetic or epigenetic regulation. Recent advances in LLPS may provide a new framework for understanding the relationship between mutation and CSCs. Studies have shown that key residue mutations in different amino acids interfere with AKAP95 condensation in opposite directions. Importantly, the activity of AKAP95 in the regulation of splicing is abolished by disruption of the condensate, significantly impaired by hardening of the condensate, and restored by replacing its condensation-mediated region with other condensation-mediated regions of an unrelated protein. Furthermore, the ability of AKAP95 to regulate gene expression and support stem tumorigenesis requires that AKAP95 forms condensates with appropriate mobility and dynamics (27). These results link phase separation to tumor stemness and may provide opportunities for therapeutic intervention in cancer.

In cancer, epigenetic modifications (DNA methylation, histone modification, chromatin remodeling, etc.) play an important role in the ‘inhibition or activation’ of different genes, especially in maintaining the stemness of CSCs (28, 29). The recognition of epigenetic modifications (30), including the interaction of transcription factor complexes with DNA sequences and the occurrence of autophagy (31), was formed accompanying phase separation. CSCs are characterized by dysregulation of diverse cellular processes, which have been the subjects of detailed genetic, biochemical, and structural studies (32). However, only recently has evidence emerged that many of these processes are formed by LLPS, which compartmentalizes protein and RNA molecules with related functions.

Therefore, it is salient to explore the relationship between phase separation and CSCs. In this review, we first summarized the key phenomenon and experimental detection of phase separation. Second, we elucidated the role of phase separation in regulating epigenetics and CSCs, which provided insights into the molecular mechanism underlying the pathogenesis of various diseases. Third, regulating phase separation is a good strategy for inhibiting the stemness of CSCs, thus providing an approach for CSC-targeted tumor therapies.

## Phase separation phenomenon and experimental detection

The phase-separated proteins initiate, amplify, and propagate signals efficiently, primarily because they have been brought into proximity and are at high density. Phase separation is now recognized as a fundamental biological mechanism wherein distinct activated molecules assemble into a different phase from the neighboring constituents of a cell. This process is important in many reactions, including but not limited to signaling and transcription. These include the assembly of adaptors after T-cell receptor (TCR) signaling, the activation of cytosolic receptors for nucleic acids, and the formation of inflammasomes. The observation of phase separation in intracellular cells is a discovery that has important implications for the study of the function of membrane-less organelles in regulating cells.

### Multivalent interaction-mediated phase separation

**Figure 1:**
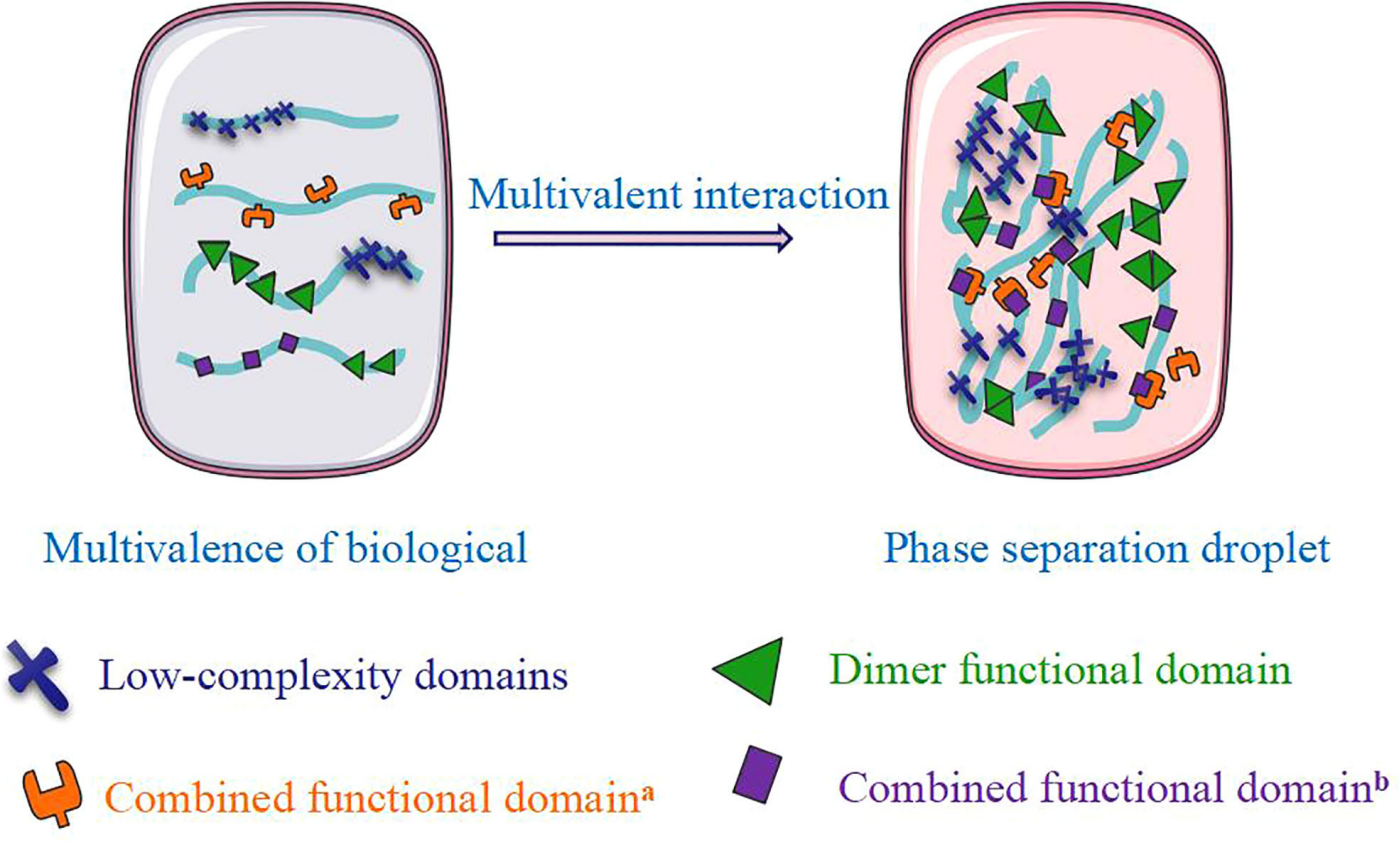
The formation of phase separation.

Numerous membraneless organelles assemble *via* LLPS, known as condensates, and facilitate the compartmentalization of cellular functions. In most cases, these structures exhibit liquid characteristics and are therefore described as bodies, puncta, granules, droplets, and condensates. We characterized condensates into three groups, i.e., plasma membrane, cytoplasm, and nuclear-localized condensates. These condensates play unexpected roles in various cellular processes (**Table 1**). Emerging evidence shows that phase separation also acts in cargo trafficking pathways by sorting and docking cargos for translocon-mediated transport across membranes, shuttling cargos through the nuclear pore complex and triggering the formation of surrounding autophagosomes for delivery to lysosomes (59, 60). Brangwynne’s group used the CasDrop system to show that the ‘LLPS’ phenomenon in the nucleus could sense and restructure the genome (37). Research on the nucleolus indicated that a dense cluster of genetic materials and proteins also showed droplet-like behaviors in the cellular nucleus (6). Phase separation driven by the multivalent interaction of molecules can form aggregates and precipitate solutions known as multivalent phase separation. This droplet formation of biological macromolecules through multivalent interaction is called LLPS (2) (**Figure 1**). Multivalent interaction refers to the process in which a multivalent ligand binds and cooperates with one or more receptors with enhanced functional affinity (apparent affinity). The multivalent interactions of intracellular biological macromolecules include the linear modification of functional domains, oligomeric proteins, and the appearance of multiple site proteins (phosphorylation, methylation, acetylation, and ubiquitination) (61). One well-known example is the proline-rich motif (PRM) and Src homologous 3 (SH3) domains, a pair of regular interacting domains existing in many intracellular proteins, forming phase separation droplets when mixed in a series of purification experiments (62). Simultaneously, the formation of droplets has a significant concentration and valence dependence. Multivalent phase separation has unique physical characteristics, including fluidity, meltability, and recovery after fluorescent bleaching. In addition to the domains with a regular structure, many proteins also contain regions without a fixed structure, called intrinsically disordered regions (IDRs) or intrinsically disordered proteins (LCDs). LCDs are usually only enriched for residue types such as Gly, Ser, Tyr, and Gln and have a strong amino acid preference and self-sustaining aggregation potential. They can also form multivalent phase separation due to the unique amino acid distribution (63, 64).

**Table 1:** Summary of evidence used for LLPS.

Localization	Compartment	Protein (s)	Refs
Cytplasm	P Granules	PGL-1	(15)
	Cytoplasm	Synthetic SH3/PRM (NCK and N-WASP)	(2)
	Stress Granule	hnRNPA 1	(33)
	Whi3 droplets	Whi-3	(34)
	Cell Stress	Pab	(35)
	Centrosome	SPD-5	(36)
	Synthetic	Opto-FUS; Opto-hnRNPA1; Opto-DDX4	(37)
	P Granules	PGL-1	(38)
	Innate immune	cGAS	(39)
	Cell stres	Sup35	(40)
	Synthetic	Synthetic FUS fusion	(41)
	P Granules	MEG-3; PGL-3	(42)
Nucleus	Nucleolus	–	(6)
	Nuages (granules)	DDX-4	(43)
	Cell Stress	EWS; TAF15; FUS	(44)
	Stress Granule	FUS	(45)
	–	Nephrin	(46)
	Nucleolus	NPM1; FIB1	(47)
	Splicing	TDP43	(48)
	Heterochromatin	HP1α	(49)
	Heterochromatin	HP1α	(30)
	Splicing	FUS; HnRNPA1; TDP43; EWSR1; TAF15	(50)
	Transcription	MED1; BRD4	(51)
	Transcription	RPB1; MED19	(52)
	Transcription	OCT4	(53)
	Transcription	ERα	(54)
	SPOP droplets	SPOP/DAXX	(11)
	Synthetic	Synthetic BRD4/Cas9	(5)
Nucleolus	Nucleolus	FIB-1	(55)
Plasma Membrane	Plasma Membrane	LAT	(56)
Pyrenoid	Carbon fixation	Rubisco/EPYC1	(57)
Nucleus; cytoplasm	Synthetic	Opto-FUS; Opto-hnRNPA1; Opto-TDP43; Opto-DDX4; Opto-PGL1	(58)

The interactions between proteins and proteins and nucleic acids are influenced by the biological macromolecule phase separation process. RNA-binding proteins containing IDRs can self-isolate, and RNA can enhance the phase separation of these proteins (65). It has been reported that 47-fold repeated CUG and CAG sequence RNA can be phase-separated spontaneously *in vitro*, which proves that the nucleic acid can be phase-separated by itself for the first time and supports the phenomenon of phase separation in the nucleus (66). Phase separation in the nucleus is closely related to epigenetic regulation and thus regulates cellular function. Therefore, it is urgent to detect phase separation with effective methods.

### Experimental detection of phase separation

With the development of new methods and techniques, LLPS will continue to be probed for both *in vitro* and *in vivo* experiments (**Table 2**). The most universal methods are ordinary optical microscopy *in vitro* and the ‘optoDroplet’ system technique *in vivo*.

**Table 2:** Summary of experimental detection for phase separation.

Equipments/Methods	Applications	Disadvantages	Refs
Ordinary optical microscope	Visualized the structure and composition of these biomolecular condensates	Condensates must be antibody-stained or fluorescently labeled	(3, 67)
Confocal microscopy and superresolution imaging	Detailed information on biomolecular condensates		(13, 68)
FRAP, FLIP, and FCS	Known to the fluidity of the condensates	Composition, concentration, and function of these biomolecular condensates are not well understood	(69)
optoDroplet	Known to the role of condensates in promoting biological function or dysfunction	Difficult operation and expensive	(37)

Fluorescence recovery after photobleaching (FRAP); Fluorescence loss in photobleaching (FLIP); Fluorescence correlation spectroscopy (FCS).

At the *in vitro* level, first, the phase-separated system has the characteristic that the solution changes from clear to turbid, forming oil droplet-like particles observed by an ordinary optical microscope *in vitro* (3, 67). Second, another droplet formation assay *in vitro* was developed to measure small-molecule partitioning into nuclear condensates and to study the behavior of small molecules within these droplets (70). The ability of MED1, BRD4, SRSF2, HP1α, FIB1, and NPM1 to form has been confirmed with this assay *in vitro* (70).

**Figure 2:**
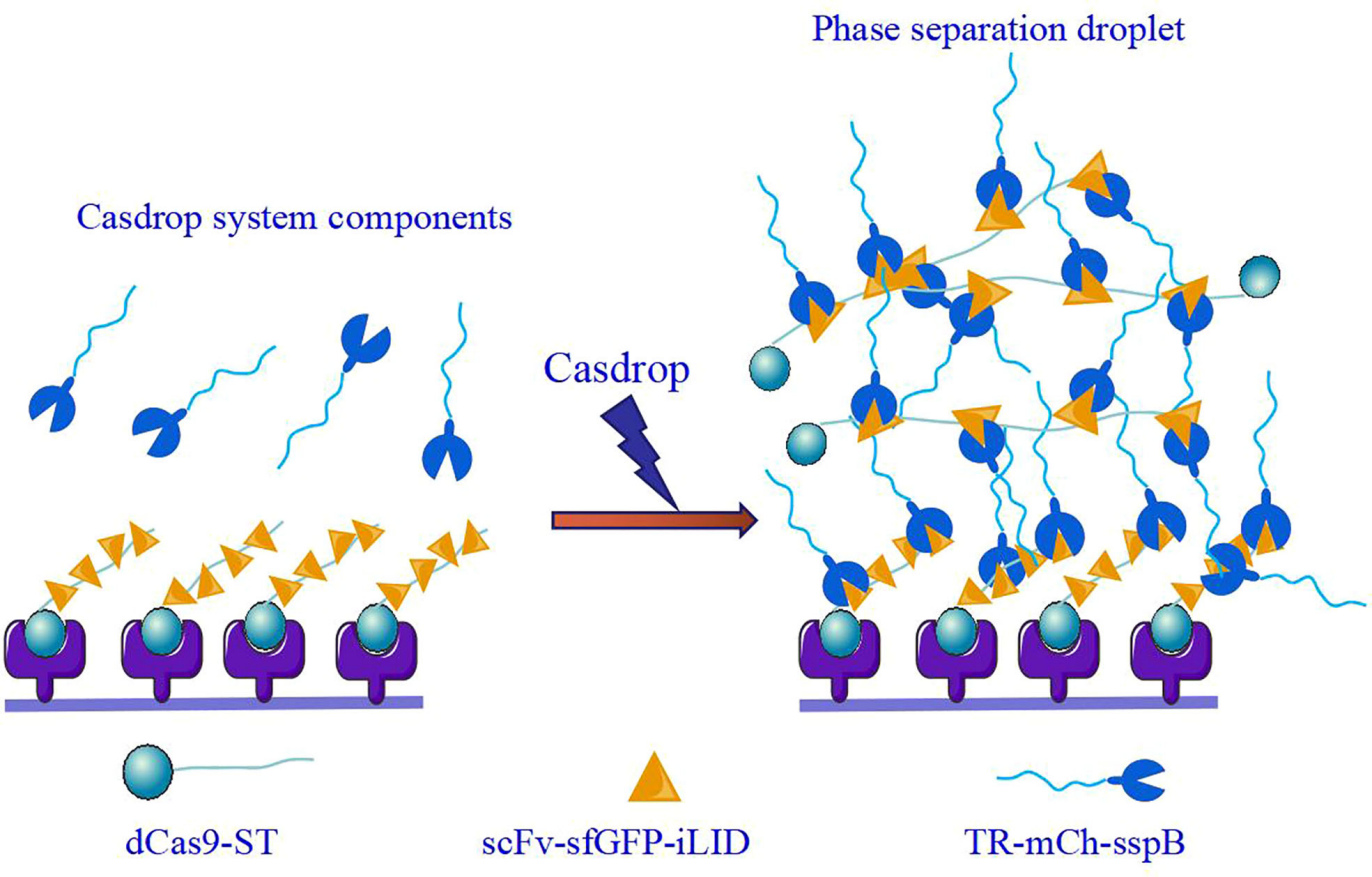
CasDrop phase separation detection system.

At the *in vivo* level, first, the widely accepted standard for phase separation detection is fluorescence recovery after photobleaching (FRAP). The FRAP method can be generally used to measure the mobility of proteins inside the cell or its organelles by calculating the diffusion coefficient of green fluorescent protein (GFP) and some other fluorescent proteins in the cells (5). Therefore, FRAP can verify the speed at which the target protein recovers fluorescence in a short period and determine whether the active area has a frequent material exchange with the surrounding environment. Second, the controllable phase separation system is the ‘optoDroplet’ system reported by Brangwynne et al. in 2017. This showed that concentrated phases are driven by IDR of various RNA/protein (RNP) human proteins, such as the fusion of sarcoma (FUS), dead-box helicase 4 (DDX4), and heterogeneous nuclear ribonucleoprotein A1 (HNRNPA1) (37). To investigate phase separation in the nucleus, researchers have explored a photo genetic platform called ‘CasDrop’ that can induce localized condensation of droplets at specific genomic sites (5) (**Figure 2**). CasDrop includes dead Cas9 (dCas9) fused with SunTag (dCas9-ST) (6), a single-chain variable fragment (scFv) fused with superfolder GFP (sfGFP), the optogenetic dimerization protein iLID (scFv-sfGFP-iLID) (15) and TR-mch-sspB. The dCas9-ST protein anchors the system to specific genetic loci (71); scFv-sfGFP-iLID protein is a photosensor polymer, and the target protein IDRs are labeled sspB. The CasDrop system added genome-targeting programmability and optogenetic controllability. This system can quantitatively and locally study the phase separation of multiple proteins. These techniques contribute to the role of phase separation in gene expression and epigenetics in cells. What are the mechanisms of phase separation in regulating gene expression?

## Mechanism of phase separation formation by posttranslational modifications

Both protein translation modifications (PTMs) and epigenetic regulation are important for protein modification and function. PTMs refer to chemical modification by adding different chemical groups to the amino acid residues of proteins (72). Phase separation is involved in the formation of PTMs, which keeps proteins with a certain function.

### Phase separation and protein translation modifications

PTMs play an important role in regulating protein function and phase separation formation in eukaryotes. PTMs include multiple modification mechanisms, such as methylation, acetylation, phosphorylation, adenylation, ubiquitination, and ADP-nucleoglycation. Among them, serine/tyrosine phosphorylation, arginine methylation (43, 73), and sulfonylation (74) have been reported to control the phase separation process (75). For example, Tau phosphorylation (p-tau217 and p-tau181), a hallmark of the pathology Alzheimer’s disease (AD) (76), promotes aggregation and phase separation *in vitro* (77).

However, protein phosphorylation also inhibits phase separation. Autophagosome and proteasome formation in the autophagy process. Mammalian target of rapamycin complex 1 (mTORC1) inhibits the droplet-like formation of Atg13 by inducing Atg13 phosphorylation at the ser428/9 site (4). Under acute hyperosmotic pressure, phase separation mediates the formation of proteasomes that promote the degradation of ribosomal and misfolded proteins. The proteasome inhibitors MG-132 and b-AP15 and the p97 inhibitor NMS-873 can inhibit the formation of phase separation simultaneously (78). Dual-specificity tyrosine phosphorylation-regulated kinases 3 (DYRK3), the human homolog of MBK-2, also induces the dissolution of several membraneless organelles during mitosis, indicating that PTMs are important for the assembly and disassembly of P granules mediated by phase separation (79).

### Phase separation and epigenetic modification

Phase separation is also involved in epigenetic regulation that maintains the stability of the organism’s genome. Epigenetic regulation modulates subsequent gene expression and participates in a variety of biological processes, mainly methylation and histone modification (methylation, acetylation, phosphorylation, etc.), and chromatin remodeling (80, 81).

#### Methylation

Both DNA methylation and RNA methylation are important nucleic acid modifications in gene expression regulation and biological processes. Methyl-CpG-binding protein 2 (MeCP2) is a chromatin organizer. MeCP2 induces compaction and LLPS of nucleosomal arrays *in vitro* and further enhances the formation of chromatin condensates by DNA methylation. The results identified a novel mechanism by which phase separation underlies MeCP2-mediated heterochromatin formation (82).

Reversible RNA methylation modification (N-methyladenosine (mA)) is the most common nucleotide modification in mRNA and is involved in various processes of mRNA metabolism, including but not limited to posttranscriptional splicing, translation efficiency, and regulation of mRNA stability (83, 84). Recent studies demonstrated that the cytosolic mA-binding protein YTH N6-methyladenosine RNA binding protein 1 (YTHDF1) and its siblings YTHDF2 and YTHDF3 undergo LLPS *in vitro* and *in vivo* (85). The number and distribution of mA sites can influence transcriptome composition during cell phase separation. Incubation with LC^+^ YTH protein showed that 50 nt RNA with more m6A (10) modifications could significantly promote phase separation, but m6A (1 or 5)-modified RNA had no significant effect on phase separation (86, 87). Therefore, phase separation regulated the cellular characteristics that were modified by mA mRNA.

#### Phase separation and histone modifications

Eukaryotic chromosomes are enriched with specific histone modifications. Constitutive heterochromatin is a largely silent chromosome compartment, partly characterized by H3K9me2 and 3. Heterochromatin protein 1 (HP1) (H3K9me2 and 3’reader’) interacts with SUV39H1 (H3K9me2 and 3 ‘writer’) and forms complexes with H3K9me2 and 3-modified chromatin. The H3K9me2- and H3K9me3-labeled nucleosome arrays and related complexes undergo phase separation by forming droplets that are rich in macromolecules, resulting in the regulation of the general mechanism of chromosome compartmentalization. These droplets are reminiscent of heterochromatin, as they are dense chromatin-containing structures, are resistant to DNase, and exclude the general transcription factor II B (TFIIB) (88). Although LLPS of HP1α is known to contribute to heterochromatin organization, another study has shown that histone H1 condenses into liquid-like droplets in the nuclei of HeLa cells and then serves as a scaffold for the separation of heterochromatin domains from DNA (89). Therefore, phase separation regulates cellular characteristics associated with the condenses and segregation of HP1.

#### Phase separation and chromosome remodeling

Chromatin remodeling is another important epigenetic regulatory mechanism. By forming remodeling complexes, the entanglement density of chromatin is changed, which affects the binding of transcription factors with DNA sequences and further regulates gene expression (90). The advent of high-throughput genome sequencing technology, such as chromatin immunoprecipitation sequencing (ChIP-seq) (91, 92), high-throughput chromatin conformation capture (Hi-C) (93, 94), and assays for transposase accessible chromatin (ATAC-seq) (95, 96), have promoted the rapid development of chromatin three-dimensional (3D) structures. Phase separation not only largely accumulates in the nucleus but also has an important relationship with the spatial structure of chromatin. In 2019, Gibson et al. reported that purified tandem nucleosomes could form phase separation with high efficiency with the participation of histone H1 *in vitro*. Since tandem nucleosomes are the most basic structural unit of chromatin, the formation of finer chromatin structures is likely to occur on this phase-separated aggregate (8). *Via* LLPS, HP1α protein forms a liquid-phase stable compartment, which contains chromatin and repels molecules such as RNA polymerase, leading to the first step in the formation of heterochromatin (30).

Phase separation and chromatin remodeling of the brominated polyglycoprotein 4 (BRD4), FUS, and TATA-box binding protein associated factor 15 (TAF15) proteins were observed with the Casdrop system. Studies have shown that Drosophila HP1a protein undergoes liquid–liquid demixing *in vitro* and nucleates into foci that display liquid properties during the first stages of heterochromatin domain formation in early Drosophila embryos. Furthermore, in both Drosophila and mammalian cells, heterochromatin domains exhibit dynamics that are characteristic of liquid phase separation, including sensitivity to the disruption of weak hydrophobic interactions, reduced diffusion, increased coordinated movement and inert probe exclusion at the domain boundary. The result shows that heterochromatic domains form *via* phase separation and mature into a structure that includes liquid and stable compartments (30). Mao et al. described nonmembranous structures in the nucleus, such as the nucleolus, Cajal body, and promyelocytic leukemia (PML), which tended to form in low-density chromatin regions (97). To further study why droplets tend to repel chromatin that is preferentially generated in regions with low chromatin density, Shin et al. established a mathematical model of the mechanical interaction between aggregates and deformable chromatin networks and suggested that all regions of chromatin density could form small droplets. However, when the droplets were enlarged to a size resolvable by an optical microscope, they tended to form in regions of low chromatin density (5). These properties of phase separation are summarized as a chromatin filtration model; that is, the phase separation of proteins binding to specific gene sites can shorten the distance of genes and exclude gene regions without binding sites. This ensures the accumulation of super transcription factors in regions with loose chromatin structure, folding chromatin and promoting the expression of active genes.

## PTM and epigenetic modification regulate cancer stem cells

This biomolecular condensate represented by phase separation may influence epigenetics in normal cells and cancer cells, especially CSCs, a small subpopulation of cancer cells responding to the initiation maintenance metastasis and therapy resistance of cancer (98). It is well known that the characterization of CSCs is regulated by PTM and epigenetic modification.

### PTM regulates cancer stem cells

PTMs play an important role in cell signal transduction. For example, epidermal growth factor (EGF) is essential for the maintenance and growth of GSCs (99). Quantitative phosphoproteomic analysis of EGF-stimulated GSCs was performed to acquire network-wide information on the molecules related to stemness maintenance. As a result, a total of 6073 phosphopeptides from 2282 phosphorylated proteins were identified, leading to the quantitative classification of 516 upregulated and 275 downregulated phosphorylation sites (100, 101). After quantitatively analyzing the proteome and phosphorylated proteome of 45 medulloblastoma samples, Archer et al. showed that tumors had a similar level of RNA expression but differed significantly at the posttranscriptional and posttranslational levels. The posttranslational modification of MYC was found to be associated with poor prognosis in group 3 tumors. Currently, many studies are focused on proteomics, which can provide a more comprehensive functional reading for future therapeutic strategies (102).

### Epigenetic regulation of cancer stem cells

**Figure 3:**
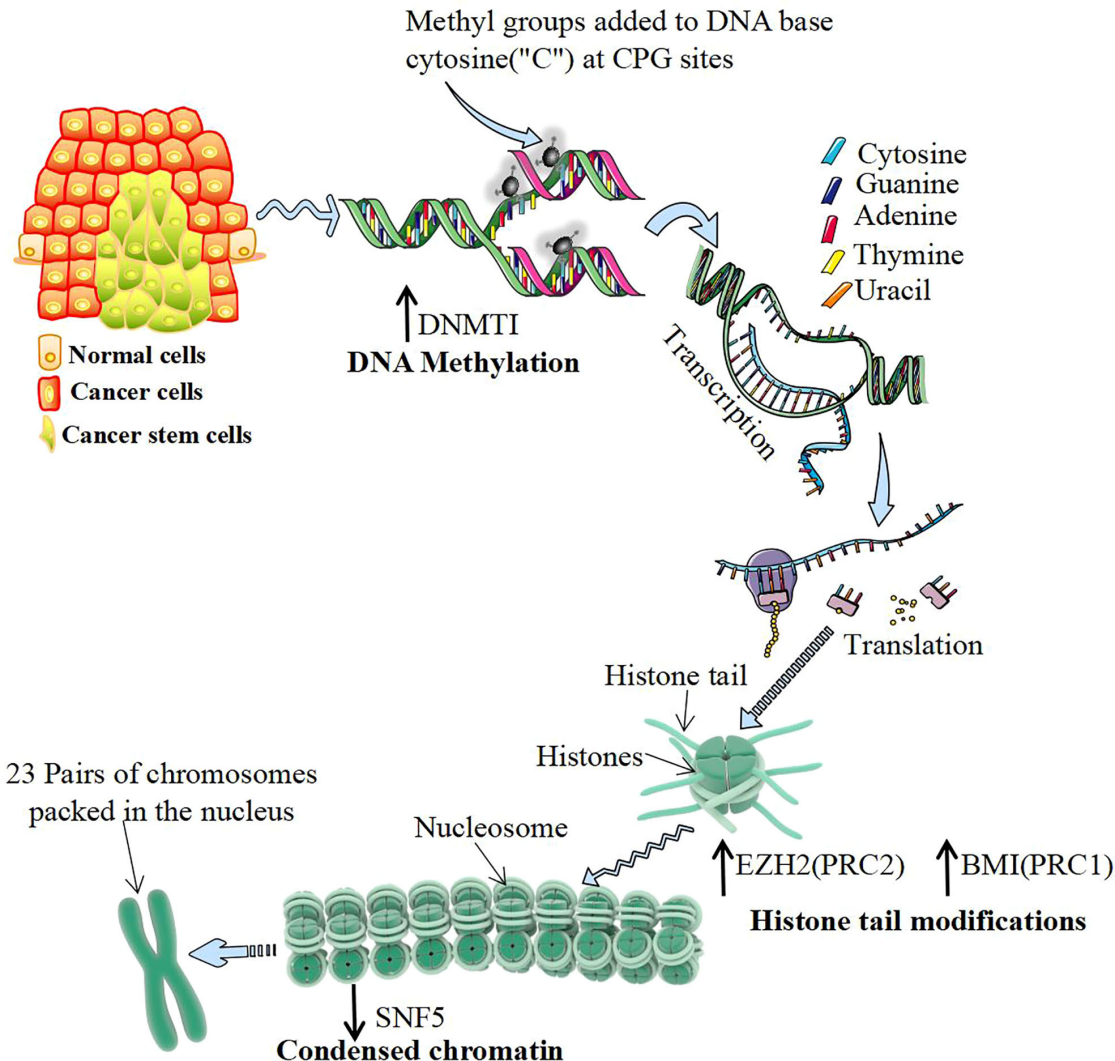
Epigenetic regulation of cancer stem cells. DNA methylation mostly occurs in CG-rich gene regions, which are catalyzed by DNA methyltransferase 1 (DNMT1). DNMT1 regulates DNA methylation and de novo synthesis of enzymes, which is the key to maintaining the characteristics of cancer stem cells (CSCs). Histone modification often occurs at the amino end of the histone, which is exposed to chromatin and can be modified by various chemical groups. Polycomb group proteins (PcGs) are important proteins catalytically inhibitory to histone modification, mainly by polycomb repressive complex 1 (PRC1) and PRC2 composition. The activity of PRC2 subunits from H3K9me and H3K27me EZH2 catalytic histones and the activity of PRC1 subunits BMI1 catalyze histones to form H2A ubiquitination. Both of them play the role of silencing genes and upregulating their expression levels, which can be directly detected with the increase in CSCs. Chromatin remodeling is another important epigenetic regulatory mechanism. The chromatin remodeling protein SNF5 can alter the DNA conformation by interacting with the promoter region of the target gene. Inactivating SNF5 or reducing its expression can promote the proliferation of CSCs.

The occurrence of CSCs has different regulatory mechanisms, but most of them are closely related to epigenetic processes (90) (**Figure 3**). DNA methylation of specific genes regulates the proliferation and differentiation of progenitor cells and is the basis of the stemness of CSC expression. Knockout of DNA methyltransferase 1 (DNMT1) maintains the characteristics of CSCs by maintaining the cell proliferation capacity and inhibiting differentiation. The m6A demethylase AlkB homolog 5 (ALKBH5) maintains the tumorigenicity of GBM stem-like cells by maintaining forkhead box m1 (FOXM1) expression and the cell proliferation program (103). Histone modification regulates the stemness of CSCs. EZH2 inhibits DACT3 and activates the Wnt/b-catenin pathway to maintain the self-renewal ability of CSCs (104). Knocking out the BMI1 gene inhibits the cloning and sphere formation ability of CSCs (105). Studies have shown that abnormal levels of the histone demethylases lysine demethylase 6A (KDM6A) and KDM6B are associated with pediatric acute myeloid leukemia (AML) (106). Moreover, modification of histone proteins (H3K9ac, H3K27ac, H4K16ac, *etc.*) play a key role in the progression and prognosis of head and neck squamous cell carcinoma (107). Chromatin remodeling changes the density of chromatin entanglement and then affects the binding of transcription factors to DNA sequences. In human malignant rhabdomyosarcoma, inactivation of SNF5 enhances Gli expression and promotes the proliferation of CSCs (108). The role of the 3D genomic structure in guiding the functional characteristics of GSCs shows that CD276 is located in a structurally conserved region of GSCs and is also part of its stemness network and could be targeted with an antibody−drug conjugate to curb self-renewal (109).

## Phase separation regulates cancer stem cells by epigenetic modification

First, phase separation is closely related to stem cell differentiation by modulating the cell cycle process. The DYRK3 enzyme promotes the mixing of phases when the cell divides, ensuring that the chromosomes separate and the cell contents divide correctly. If the phase separation is abnormal during cell division, the separation of chromosomes is incomplete, and they may be incorrectly assigned to daughter cells (79). Phase separation is an embodiment of epigenetic regulation that regulates the self-renewal of CSCs (110). Speckle-type POZ protein (SPOP), located in the nucleus, is the ligand of the substrate of cullin-3 cycloubiquitin ligase. The cancer-related SPOP mutation interferes with the recruitment of ligase substrates, leading to the accumulation of proto-oncoproteins, triggering phase separation of SPOP and colocalization of membrane-free organelles in cells. The substrate phase separation of the E3 ligase is the basis of ubiquitin-dependent protein stable ubiquitination (11). It can be speculated that phase separation promotes cancer occurrence through ubiquitination and might promote the tumorigenicity of CSCs.

Second, protein phase separation could impact chromosome remodeling. In both Drosophila and mammalian cells, a heterochromatin formation model suggested that the LLPS of HP1α protein might be the result of the increased binding degree of HP1α to chromatin polymer (30). Specifically, HP1α forms small droplets through phase separation, which ‘seals’ a specific genome, resulting in the target genes being silenced. Studies have confirmed that protein phase separation is involved in cellular structure and helps to maintain genome stability. Furthermore, mutational analyses have revealed a finer, innate compartmentalization in Hi-C experiments that likely reflects contacts involving smaller domains/complexes. Proteins that bridge (modified) DNA and histones in nucleosomal fibers. The HP1α-H3K9me2/3 interaction represents the most evolutionarily conserved paradigm that could drive and generate the fundamental compartmentalization of the interphase nucleus. This has implications for the mechanism that maintains cellular identity to be a terminally differentiated fibroblast or a pluripotent embryonic stem cell. Furthermore, HP1α plays an important role in the formation and function of CSCs (49). Tau protein plays an important role in the biology of stress granules and the stress response of neurons. The results show that the interaction of tau phosphorylation with RNA and the RNA-binding protein TIA1 is sufficient to drive phase separation of tau at physiological protein concentrations, without the requirement of artificial crowding agents such as PEG. Using this system, they further demonstrated that TIA1 also promotes tau oligomerization and vitrification. Interestingly, they find that TIA1 exhibits a selective ability to copartition with tau under physiological conditions, which speaks to the importance of TIA1 in tau biology. Finally, they observe that the tau produced by *in vitro* interactions with TIA1 and RNA is highly neurotoxic, unlike other conformers of tau produced *in vitro*. The discovery will help advance drug development to screen for potential compounds that prevent the formation of tau oligomers (111).

Finally, the phase separation process had a prominent role in the formation of the 3D genome conformation (112). Advances in 3D chromatin folding technology have made genome conformation play a prominent role in transcriptional regulation. Characterizing genome structures has profound implications for cancer (113). In adult glioblastoma, a new relationship between 3D genome architecture and stemness properties in GSCs has been reported. In particular, by integrating multiple genomics and structural genomics data sets, it was discovered that there is an unexpected link between immune-related genes and self-renewal programs in GBM. CD276 is part of a stemness network in GSCs and can inhibit self-renewal through antibody−drug conjugates. The 3D genome structure could guide the functional characteristics of GSCs, and CD276 inhibitors could decrease the GSC population in GBM (109).

In the future, phase separation may regulate CSCs through histone modification, chromosome remodeling, and 3D genome architecture. Phase separation may weaken the tumorigenicity of CSCs by regulating epigenetic modification, thereby inhibiting the occurrence of cancer, and may achieve therapeutic tumorigenesis.

Various types of therapeutic strategies targeting CSCs have been developed, including targeting cell surface markers, signal transduction pathways, the microenvironment, and metabolic patterns of CSCs, as well as other strategies, such as pro-CSC differentiation and immunotherapy targeting CSCs. A monoclonal antibody targeting CD133 exhibited a significant killing effect on CD133+ GSCs (114). Many studies on the Notch pathway in CSCs have shown that activation of Notch promotes cell survival, self-renewal, and metastasis and inhibits apoptosis. Aberrant Notch signaling (Notch1 and Notch4) promotes the self-renewal and metastasis of breast and hepatocellular carcinoma stem cells (115, 116). Sox2 belongs to the family of high-mobility group transcription factors and is also a key transcription factor in maintaining the self-renewal ability of tumor-initiating cells (TICs). Knocking out Sox2 inhibits glioblastoma cell proliferation and tumorigenicity (117). Inhibiting Sox2 also decreased metastasis in invasive cutaneous squamous cell carcinoma (SCC) (118).

Therefore, phase separation plays an important role in regulating the stem cells of CSCs, and regulating phase separation may become a potential strategy for targeted therapy of CSCs. The phenomenon of phase separation in cells has only discussed ‘structural encapsulation’ and ‘selective enrichment and rejection’ but cannot explain how phase separation is regulated in cells. Answering this question will help us clarify the mechanism of phase separation in tumor formation. Then, we can precisely block phase separation as potential tumor targets. Thus, more investigation and a greater understanding of phase separation are needed to generate novel therapeutics for cancer.

## Conclusions and perspectives

Phase separation does provide an attractive model by which to explain the division of the nucleus and the regulation of the many different biochemical reactions that take place in the nucleus. Although most of the liquid condensates described above were identified many years ago, it has only now become possible to mechanistically dissect their dynamics during different biological processes, ranging from the transcriptional to the translational level. However, an increasing number of questions have also emerged. For example, what is the underlying mechanism in the regulation of biomolecular condensates by their material properties? How do disease-associated mutations or PTMs regulate the physical properties of condensates? How to regulate LLPS to achieve the desired therapeutic CSCs remains to be explored. Overall, although the field of LLPS is young and rapidly developing, this mechanism has undoubtedly revolutionized our understanding of various biological activities and tumor disease conditions. It is expected that basic research in LLPS and oncological diseases will continue to be refined and translated into clinical practice.

## Author contributions

CX, HZ, GC, GW, and PC participated in the design of this study and organized the manuscript. CX wrote the draft of the manuscript. HZ, GC, and LG designed and revised the manuscript. All authors contributed to the article and approved the submitted version. We thank Dr. Oscar Junhong Luo and Pengcheng Wang for the critical proofreading of the manuscript and suggestions for improvements. All authors contributed to the article and approved the submitted version.

## Funding

This work was supported by grants from the Natural Science Foundation of Guangdong Province (2019A1515011966, 2021A1515220144) and the National Natural Science Foundation of China (82073014).

## Conflict of interest

The authors declare that the research was conducted in the absence of any commercial or financial relationships that could be construed as a potential conflict of interest.

## Publisher’s note

All claims expressed in this article are solely those of the authors and do not necessarily represent those of their affiliated organizations, or those of the publisher, the editors and the reviewers. Any product that may be evaluated in this article, or claim that may be made by its manufacturer, is not guaranteed or endorsed by the publisher.
